# New Therapeutic Avenues of mCSF for Brain Diseases and Injuries

**DOI:** 10.3389/fncel.2018.00499

**Published:** 2018-12-20

**Authors:** Vincent Pons, Serge Rivest

**Affiliations:** Neuroscience Laboratory, Centre Hospitalier Universitaire (CHU) de Québec Research Center and Department of Molecular Medicine, Faculty of Medicine, Laval University, Quebec, QC, Canada

**Keywords:** mCSF, microglia, brain diseases, innate immune response, phagocytosis

## Abstract

Macrophage colony-stimulating factor (mCSF) is a cytokine known to promote the recruitment of macrophages inducing the release of CCL2, a chemokine mobilizing monocytes to sites of inflammation. Additionally, it induces microglia/macrophage proliferation and the polarization of these cells towards a M2-like phenotype, impairing their ability to release pro-inflammatory factors and toxic mediators, while favoring the release of mediators promoting tissue repair. Another important player is the mCSF receptor CSFR1, which is highly expressed in monocytes, macrophages and microglia. Here, we discuss the new interesting therapeutic avenue of the mCSF/CSFR1 axis on brain diseases. More specifically, mCSF cascade might stimulate the survival/proliferation of oligodendrocytes, enhance the immune response as well as modulate the release of growth factors and the phagocytic activity of immune cells to remove myelin debris and toxic proteins from the brain.

## Introduction

Macrophage colony-stimulating factor (mCSF) is an hematopoietic cytokine expressed in a wide range of cells and tissues, namely the kidney, brain, liver, retina, spleen, lung, adipose tissue, skin and joints (Ryan, 2001; Nandi, 2006). It stimulates progenitor cells from bone marrow (Stanley, 1976) and takes a role in the development, proliferation and maintenance of mononuclear phagocytes such as monocytes, dendritic cells, microglia and osteoclasts (Chitu et al., 2016). mCSF signals through a tyrosine kinase family receptor, CSFR1 also known as CD115, which also binds interleukin-34 (IL-34) that plays similar roles (Ségaliny et al., 2015). In addition to be involved in the development and support of innate immune cells, mCSF/CSFR1 activity is involved in various pathologies such as ovarian cancer, breast cancer, rheumatoid arthritis and cutaneous lupus (Toy et al., 2009; Achkova and Maher, 2016). The mCSF/CSFR1 axis is a therapeutic target to regulate the inflammatory and pathological processes, including cancer, rheumatoid arthritis and cutaneous lupus. CSFR1 monoclonal antibody or antagonist has been shown to suppress the inflammatory response in a phase II clinical trial on rheumatoid arthritis (Garcia et al., 2013). mCSF-deficient mice exhibit numerous phenotypic defects, namely toothless, skeletal defects (Naito et al., 1997), reduced body weight, deficit in tissue macrophages and osteoclasts as well as neurological abnormalities (Nandi et al., 2012) indicating a major role played by this ligand on these populations of cells. In the brain this cytokine is secreted by neurons, astrocytes and microglia and is involved in the brain development (Nandi et al., 2012). In this regard, mCSF-deficient animals have severe brain deficits with specific abnormalities within the cerebral cortex.

mCSF has also a critical role on the activity, survival, maintenance, proliferation and differentiation of microglial cells, which are resident immune cells in the brain. It has been proposed that microglia are derived from progenitors that originate from the neuroectoderm and/or the mesoderm, which colonize the brain from the early embryonic stage, and throughout the fetal development stage (Soulet and Rivest, 2008). They are the only cell type that expresses the CSFR1 in the brain (Erblich et al., 2011). A conditional deletion of CSFR1 specifically in microglia leads to a severe depletion of these innate immune cells in the brain (Elmore et al., 2014). Taken together, these data point towards a major role of the mCSF/CSFR1 axis within microglia as a crucial neuroprotective mechanism in the brain during normal and pathological conditions.

### mCSF in Pathological Conditions

mCSF has the ability to polarize microglia towards both pro-inflammatory (M1) and anti-inflammatory (M2) directions depending on the microenvironment and other inflammatory molecules (Hamilton et al., 2014). The presence of IL-1, tumor necrosis factor (TNF), reactive oxygen species (ROS) and nitric oxide (NO) creates a powerful pro-inflammatory medium, whereas IL-10, arginase 1, transforming growth factor β (TGF-β) are rather anti-inflammatory and contribute to the effects of mCSF to restrict neuroinflammation in brain injuries and diseases. (Cherry et al., 2014).

Neurodegenerative diseases are associated with a robust microglial response, which is known to have both beneficial and detrimental properties depending on the disease, animal models, duration and environmental factors (Aguzzi et al., 2013). In many diseases, microglia are becoming a key target for therapeutic purposes since they can eliminate toxic elements from the brain and set the conditions for repair and remyelination. The mCSF/CSFR1 axis is consequently a very interesting new therapeutic avenue and has also been used as diagnostic tools in neuropathologies (Hume and MacDonald, 2012). In this regard, low levels of mCSF were measured in patients with presymptomatic Alzheimer’s disease (AD) or mild cognitive impairment (MCI), which together with low levels of other hematopoietic cytokines predicted the rapid evolution of the disease toward a dementia diagnosis 2–6 years later (Ray et al., 2007; Laske et al., 2010). In multiple sclerosis (MS) patients, despite the tremendous increase in macrophages/microglia within the lesions, the relative number of these cells expressing mCSF or CSFR1 decreased (Werner et al., 2002). Consequently, a lower level of mCSF/CSFR1 in the bloodstream and the brain seems to be predictive a the disease evolution.

### Alzheimer’s Disease (AD)

AD is a progressive neurodegenerative disease and the most common form of dementia with close to 50 million affected individuals in the world. One proposed hypothesis of the disease onset and progression is the failure of microglia to clear amyloid-beta (Aβ) due to their poor phagocytic properties when compared to macrophages. The consequence is the Aβ accumulation (both soluble and insoluble) that provokes neurological disorder, cognitive decline and neurodegeneration (Masters et al., 2015). On the other hand, the harmful role of microglia is due to the production of pro-inflammatory cytokines, NO and ROS among other secreted factors in presence of Aβ (Combs et al., 2001). Although microglia have ability to clear Aβ at the early stage of AD, this phagocytic response seems to deteriorate with time explaining the overflow of Aβ accumulation while the diseases is progressing (ElAli and Rivest, 2016). Such impaired role of microglial cells to clear this toxic protein appears crucial in AD etiology. As mentioned, mCSF plays important roles in the activation state of microglia, especially to improve their phagocytic properties with very limited pro-inflammatory activities (Mitrasinovic et al., 2003). Intraperitoneal weekly injections of mCSF beginning before the apparition of symptoms prevent amyloid burden and neurological decline in the APPswe/PS1 mouse model of AD (Boissonneault et al., 2009). Even more interesting is the fact that such a treatment was able to stabilize the disease then improve the cognition and memory when started late after the first symptoms. The main conclusion of this study was that mCSF was able to trigger microglia proliferation, improve their phagocytic activities to Aβ and prevent its toxicity to neuronal elements (Boissonneault et al., 2009).

In another study supporting the neuroprotective role of mCSF, Mitrasinovic and colleagues used microglial cell line overexpressing CSFR1 in the presence of Aβ in the culture medium (Mitrasinovic, 2003). Such a preparation was able to activate microglia and improve Aβ phagocytosis. One of the challenges is to control the chronic inflammatory reaction, which has frequently been associated with the detrimental role of these cells in that disease. Of interest is that such a response does not take place in the brain of mCSF-treated animals such as in the case of lipopolysaccharide (LPS)-treated mice that exhibit of strong and robust pro-inflammatory reaction together with an increased phagocytosis. Of note, a chronic systemic LPS administration in APP_swe_/PS1 mice leads to higher Aß deposit (Lee et al., 2008). In contrast, Michaud et al. (2013) used a detoxified LPS called monophosphoryl lipid A (MLP) that triggers a low inflammatory response while inducing a strong phagocytic microglial reaction in APP_swe_/PS1 mice and improvement of AD-related pathologies. This provides clear evidence that a strong inflammatory response by microglia is not necessary to improve their efficiency to clear Aβ. The challenge remains to mastering the inflammatory/anti-inflammatory phenotype depending on stage of AD and to maintain efficient responses to clear Aβ in a chronic manner to prevent or delay the symptoms.

### Multiple Sclerosis (MS)

MS is an autoimmune disorder with consequences as axon demyelination and chronic inflammation of the CNS (Thompson et al., 2018). The demyelinating processes leave many myelin debris, which can be cleared by microglia and infiltrating macrophages later in the pathology (Huizinga et al., 2012). A proper activation of microglia enhances myelin clearance and allows the oligodendrocyte precursor cell (OPC) differentiation and oligodendrocyte survival, that improve remyelination (Kotter, 2006). As for the case of amyloid, microglia need to be activated to phagocyte the myelin debris and we recently provided solid evidence that mCSF plays a key role in such activation process (Laflamme et al., 2018). A number of animal models are available to mimic MS, namely cuprizone-induced demyelination and Experimental Autoimmune Encephalomyelitis (EAE). The well-characterized, very reproducible and non-invasive cuprizone model allows to study cellular and molecular mechanisms involved in the demyelination (DM)/remyelination (RM) process, while excluding the autoimmune component (Denic et al., 2011). Indeed, continued exposition to dietary cuprizone, a copper-chelating toxin, leads to oligodendrocyte apoptosis and demyelination among vulnerable brain structures, such as the corpus callosum and cerebral cortex. Few days after replacing cuprizone by normal food, RM is observed in those structures (Matsushima and Morell, 2006; Gudi et al., 2014). Our previous study brought us to deepen the role of microglia and other parenchymal cells such as oligodendrocytes in the cascade of events that leads to DM/RM events (Lampron et al., 2015). For this reason, in this study, exogenous mCSF was administered to mice that received dietary cuprizone (Laflamme et al., 2018). mCSF is a cytokine well-known to stimulate cell survival, proliferation and differentiation of myeloid cells (Hamilton, 2008; Otero et al., 2009). Moreover, it modulates microglial phenotype towards an anti-inflammatory one (Ushach and Zlotnik, 2016), reducing the expression of antigen presenting proteins (Smith et al., 2013) and promoting the release of trophic factors (Smith et al., 2013). Herein, we investigated the effect of exogenous mCSF on the activity of microglia and oligodendrocytes over the course of cuprizone intoxication. Since mCSF is expressed constitutively, we utilized a conditional model in which its mCSF receptor (CSF1R) is deleted in microglia selectively, to better understand the role of the endogenous cytokine.

mCSF-treated mice exhibited reduced myelin loss during the demyelination phase, together with an increased number of microglia and OPCs in lesion sites (Laflamme et al., 2018). Tamoxifen-induced conditional deletion of the mCSF receptor in microglia from cuprizone-fed mice caused aberrant myelin debris accumulation in the corpus callosum and reduced microglial phagocytic response. mCSF therefore plays a key role in stimulating myelin clearance by brain innate immune cells, which is a prerequisite for proper remyelination and myelin repair processes. Microglial cells synthetize the growth factor IGF-1 that may be involved in the remyelination process. IGF-1 would play a role in oligodendrocyte survival and OPC differentiation, but the mechanism remains unclear and needs to be further investigated (Laflamme et al., 2018).

Although the cuprizone model allowed us to study the de/remyelination and the interactions between microglia, oligodendrocytes and the environment, it is not a model to study the inflammation and peripheral immune cell invasion. For this aspect of the disease, EAE is used. In this model, there is a chronic inflammatory response together with a robust activation of microglia, DM, synaptic dysfunctions and perturbation in the axonal transport (Rasmussen et al., 2007). mCSF inhibition in EAE animals improved recovery, suppressed the production of pro-inflammatory molecules and decreased the number of infiltrating immune cells (Uemura et al., 2008). The dual role of the cytokine in these two different models may be explained by critical contribution of infiltrating cells in the pathology of EAE mice. Although there is also infiltration of monocytes in the brain of cuprizone fed mice, these cells do not contribute to the neuropathology of cuprizone-treated animals (Lampron et al., 2015). Consequently, inhibiting mCSF function seems beneficial in a model that depends on blood brain barrier damages and massive infiltration of immune cells, whereas it is the opposite in a model depending on the ability of microglia to clear myelin debris, such as in the case of cuprizone.

### Glioma

Glioma is a tumor that begins within the population of glial cells in the CNS. The tumor-associated cytokines are correlated with the grade of the cancer (Balkwill and Mantovani, 2001). In high-grade gliomas, macrophages with an anti-inflammatory profile are predominant. mCSF, IL-10, TGFβ are reported to be highly expressed by the tumor and its microenvironment compared to low grade gliomas (De et al., 2016). These results suggest that tumor-derived mCSF induces a shift of microglia/macrophages towards the M2 phenotype, which influences tumor growth. Evaluation of the proportion of M2 microglia/macrophages and mCSF expression in tumor tissue would be useful for the assessment of microglia/macrophage proliferative activity and the prognosis of patients with gliomas. Injecting mCSF in rats bearing tumors has two effects depending on the dose. A low dose of mCSF has no effect regarding the tumor progression and cell recruitment, whereas an high dose of mCSF has a significant anti-tumor effect against glioma (Matsuoka et al., 1994). Similar anti-tumor effects were reported in other cancers, such as sarcoma, melanoma and lung cancer. Another study in mCSF-deficient mice (mice op/op) has shown a comparable number of Iba1 cells in tumor-bearing brain and a comparable state of activation in both wild type and op/op groups of mice (Sielska et al., 2013). Inhibition of mCSF signaling pathway by blocking the CSFR1 on microglia prevents the glioblastoma invasion and tumor-associated microglia (Coniglio et al., 2012). Glioblastoma cells from human patient express mCSF which triggers microglia M2 profile that is involved in tumor progression (Komohara et al., 2008; Pollard, 2009). Despite conflicting data, it seems that inhibiting of the mCSF/CSFR1 pathway would be the angle to treat glioblastoma.

### Brain Injury

A massive proliferation and infiltration of innate immune cells takes place during CNS injury and microglia are the main effector of this response (Donat et al., 2017). Such a cellular response is mainly mediated by the release of cytokines in the microenvironment of the injury. mCSF inhibition was found to prevent significantly such a cellular movement together with microglial proliferation, an increase number of neuron death and exacerbation of the injury. These observations unravel another example of the beneficial role played by mCSF and the importance of these resident immune cells in the brain. Neuronal protection from excitotoxic and teratogen reaction followed by brain injury was also observed in organotypic co-culture media with transfected microglia overexpressing CSFR1. Microglia activation by mCSF improved neuronal survival in the co-culture *in vitro* system (Mitrasinovic, 2005). It was also shown that the cytokine is secreted by neurons in early phases then by microglia on later phases around necrosis in an *in vivo* model of focal brain injury in rats (Takeuchi et al., 2001), mCSF-induced microglial proliferation promotes tissue repair, nerve regeneration and inhibits production of pro-inflammatory cytokines (Xu et al., 2017). It is also important to mention that activated microglia are not strictly polarized into pro-inflammatory or anti-inflammatory profiles, but into a mixed phenotype resulting in a complex signaling between the actors of neuroinflammation. Indeed, gene expression analyses have unraveled such a mixed signature of M1/M2 microglia following brain injury (Rosi, 2016).

### Clinical Studies

mCSF/CSFR1 axis is a therapeutic target to regulate the inflammation and innate immune cell proliferation and differentiation that play both beneficial and detrimental roles in neuropathologic processes depending on the disease and model (Hamilton et al., 2017). Drugs were developed against the ligand or the receptor in various diseases namely cancer, rheumatoid arthritis and cutaneous lupus. It is targeted with monoclonal antibodies or antagonists, which block the action of the ligand on its binding site. It is the principal strategy used in cancer therapy. Monoclonal antibodies have also been developed against mCSF. Based on preclinical data, inhibition of the mCSF/CSFR1 axis may not be a good strategy and can be deleterious in inflammatory or neurodegenerative diseases (Hamilton et al., 2017). On the other hand, such a strategy seems to have beneficial effects on pain alleviation where microglia have to be inhibited in contrast to the diseases discussed above.

## Conclusion

**Figure 1:**
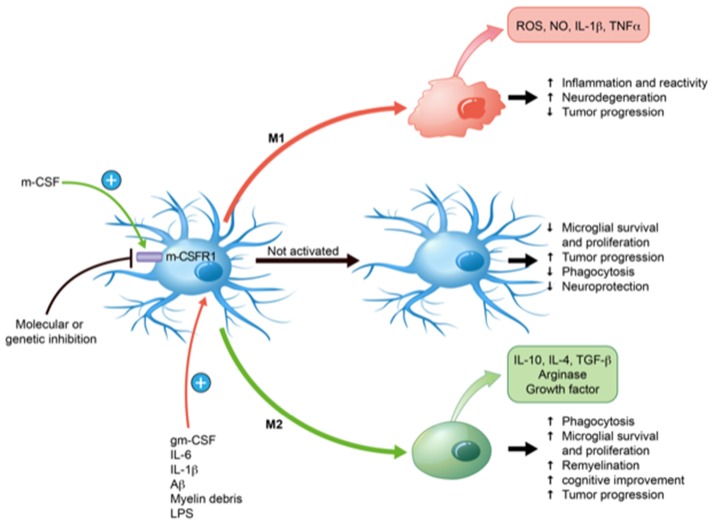
Effects of macrophage colony-stimulating factor (mCSF) on microglial polarization and functions. mCSF has the ability to polarize microglia into pro-inflammatory (M1) or anti-inflammatory (M2) depending on the environment and cytokine production. They may also remain inactivated. Their states of activation have direct consequences on their specific functions, such as phagocytosis, neuroprotection, inflammation, etc. M1 takes place in response to granulocyte-mCSF (gmCSF), interleukin-6 (IL-6), IL-1β, lipopolysaccharide (LPS), amyloid β (Aβ) and myelin debris. M1 profile shows anti-tumor progression and induces neuroinflammation with the production of reactive oxygen species (ROS), nitric oxide (NO), IL-1β and tumor necrosis factor-α (TNF-α) that may contribute to neurodegeneration. M2 profile occurs in response to mCSF, which induces remyelination, improves phagocytosis, diminishes inflammation together with the production of IL-10, IL-4, arginase, transforming growth factor β (TGF-β). These may promote neurorepair and tumor progression. Inhibition of mCSF with specific molecules or genetic construction inhibits microglia and blocking its receptor causes a marked microglial depletion.

mCSF/CSFR1 has a pivotal role in healthy brain and neurodegenerative pathologies. It is synthetized by neurons, astrocytes and microglia. Such a mCSF/CSFR1 signaling helps microglia clearing myelin debris, support neurons, clear toxic proteins and maintaining microglia (Figure 1). Many groups have studied the role of the mCSF/CSFR1 axis in the brain. It appears essential in brain development, synaptic landscape, infection resolution and neuronal maintenance. In some neurodegenerative diseases, mCSF and microglia are highly beneficial, especially in mouse model of AD to clear amyloid, prevent its toxicity to neurons and improve cognitive impairment. mCSF-activated microglia help clearing myelin debris and set the conditions for remyelination and repair. mCSF injections have beneficial effects on remyelination, oligodendrocyte survival and OPC differentiation in cuprizone-fed mice. mCSF is not always benefic, inhibition of mCSF reduces immune cell infiltration and inflammation in the EAE model that mimics the massive invasion of systemic immune cells in the CNS. In the same line, mCSF deficiency prevents tumor invasion in glioblastoma.

Taken together, these studies underline a great potential of this hematopoietic cytokine and the need to modulate the mCSF/CSFR1 axis in pre-clinical models of brain diseases, although this has to be validated in clinical settings. Many clinical trials have used mCSF inhibitors or a monoclonal antibody against CSFR1 in last years, most of them concern cancer or rheumatoid arthritis. The different effects of the molecule may be due to several factors. A better understanding of the exact role of mCSF in different neuropathologies is needed to develop strategies for the use of mCSF as new therapeutic perspectives. Different time points in the disease progression as well as the model used clearly influence the effects of the cytokine. In addition, some findings have demonstrated physiological differences between young and elderly humans, which may depend on the unalike characteristics of microglia in young and old animals. As mentioned, CSFR1 conditional gene deletion mice are quite useful to study the role of the receptor in neuropathologies and these needed studies will certainly help investigating the role of the mCSF/CSFR1 axis in clinical settings.

## Author Contributions

VP and SR wrote the manuscript together.

## Conflict of Interest Statement

The authors declare that the research was conducted in the absence of any commercial or financial relationships that could be construed as a potential conflict of interest.
